# Development and Test of a Portable ECG Device with Dry Capacitive Electrodes and Driven Right Leg Circuit

**DOI:** 10.3390/s21082777

**Published:** 2021-04-15

**Authors:** Alessandro Zompanti, Anna Sabatini, Simone Grasso, Giorgio Pennazza, Giuseppe Ferri, Gianluca Barile, Massimo Chello, Mario Lusini, Marco Santonico

**Affiliations:** 1 Unit of Electronics for Sensor Systems, Department of Engineering, Campus Bio-Medico University of Rome, 00128 Rome, Italy; a.sabatini@unicampus.it (A.S.); g.pennazza@unicampus.it (G.P.); 2 Unit of Electronics for Sensor Systems, Department of Science and Technology for Humans and the Environment, Campus Bio-Medico University of Rome, 00128 Rome, Italy; s.grasso@unicampus.it (S.G.); m.santonico@unicampus.it (M.S.); 3 Department of Industrial and Information Engineering and Economics, University of L’Aquila, 67100 L’Aquila, Italy; giuseppe.ferri@univaq.it (G.F.); gianluca.barile@univaq.it (G.B.); 4 Unit of Cardiology, Department of Medicine, Campus Bio-Medico University of Rome, 00128 Rome, Italy; m.chello@unicampus.it (M.C.); m.lusini@unicampus.it (M.L.)

**Keywords:** wearable sensors and devices, wearable monitoring systems, wearable healthcare electrocardiography, wireless ECG monitoring systems, electronics for biosignals and biopotentials, electronic interfaces and embedded sensor systems for biomedical application, dry electrodes, capacitive coupling electrodes

## Abstract

The use of wearable sensors for health monitoring is rapidly growing. Over the past decade, wearable technology has gained much attention from the tech industry for commercial reasons and the interest of researchers and clinicians for reasons related to its potential benefit on patients’ health. Wearable devices use advanced and specialized sensors able to monitor not only activity parameters, such as heart rate or step count, but also physiological parameters, such as heart electrical activity or blood pressure. Electrocardiogram (ECG) monitoring is becoming one of the most attractive health-related features of modern smartwatches, and, because cardiovascular disease (CVD) is one of the leading causes of death globally, the use of a smartwatch to monitor patients could greatly impact the disease outcomes on health care systems. Commercial wearable devices are able to record just single-lead ECG using a couple of metallic contact dry electrodes. This kind of measurement can be used only for arrhythmia diagnosis. For the diagnosis of other cardiac disorders, additional ECG leads are required. In this study, we characterized an electronic interface to be used with multiple contactless capacitive electrodes in order to develop a wearable ECG device able to perform several lead measurements. We verified the ability of the electronic interface to amplify differential biopotentials and to reject common-mode signals produced by electromagnetic interference (EMI). We developed a portable device based on the studied electronic interface that represents a prototype system for further developments. We evaluated the performances of the developed device. The signal-to-noise ratio of the output signal is favorable, and all the features needed for a clinical evaluation (P waves, QRS complexes and T waves) are clearly readable.

## 1. Introduction

The use of point-of-care (POC) systems and wearable sensors for health monitoring is rapidly growing. They are used both for clinical and for remote monitoring, improving the possibility to collect health data about patients continuously and to prevent health deterioration or clinical events [1].

POC and wearable sensors are able to empower patients, providing them with the right tools to monitor themselves autonomously. Patients are able to cooperate with medical teams and contribute to their own health [2]. Moreover, remote monitoring devices prevent the hospitalization of patients, with a consequent cost reduction for the healthcare system.

Several data can be gathered using wearable sensors in order to monitor and record real-time information about patients’ health. Sensors measure physiological signs such as an electrocardiogram (ECG), an electromyogram (EMG), heart rate (HR), body temperature, electrodermal activity (EDA), arterial oxygen saturation (SpO2), blood pressure (BP) and respiration rate (RR) [3].

There is currently a growing interest in using wearable devices to monitor cardiovascular diseases [4]. According to recent statistics, cardiovascular disease (CVD) is one of the leading causes of death globally. In Europe as a whole, it is responsible for over 3.9 million deaths a year, or 45% of all deaths [5].

Over the past decade, wearable technology has gained the interest of researchers and clinicians due to the benefits that could be associated with the long-term monitoring of CVD patients, not necessarily at a hospital. Moreover, wearable devices have attracted much attention from the tech industry. Even if the primary use of commercial wearable devices is to monitor activity parameters (heart rate, step count, etc.), the sensors used are now so advanced that it is possible to also monitor physiological parameters directly related to CVD, such as heart electrical activity or blood pressure.

Among the list of features forming the wearable technology paradigm (which can be found in many review papers or textbooks devoted to this subject [6,7,8,9]), it is here important to discuss those in this paper. A wearable device, especially for medical use, should be comfortable for the patient and adequate to follow movements. If possible, it should be effectively wearable, meaning that it could be possible to wear it with clothes. It should have electrical/electronic characteristics that make it the least disturbed by interferences.

Several commercial wearable devices, such as Apple Watch (Apple Inc., Cupertino, CA, USA), Fitbit Sense (Fitbit Inc., San Francisco, CA, USA) or Withings ScanWatch (Withings, Issy-les-Moulineaux, France), are able to record a single-lead ECG using metallic dry-contact electrodes, in contrast to adhesive wet electrodes [10]. Typically, one electrode is placed on the front of the watch and can be touched with a finger, and another electrode is placed on the back, in contact with the wrist. This one-lead ECG can improve arrhythmia diagnosis; however, for the diagnosis of other cardiac disorders, such as myocardial infarction or ischemia, additional ECG leads are required [11].

Traditional 12-lead ECG is able to record cardiac potentials on the skin, with an amplitude of a few mV and frequencies in the range of 0.01–150 Hz. The measurement has been performed for a limited time with the patient at rest, laying on an examination table. Several cables and standard wet Ag/AgCl electrodes are used. This type of electrode requires proper preparation of the skin [12]. Moreover, adhesive wet electrodes are not suitable for long-term monitoring purposes because they can cause irritation and lose their conductivity over time due to drying out [13]. As a consequence, dry-contact electrodes represent an alternative to wet contact electrodes [14], in particular for long-term monitoring, where they are more comfortable for the patient [15,16]. In recent years, noncontact electrodes have been used in several studies [17]. In particular, capacitive electrodes, coupled with the patient’s body, have been investigated [18,19,20,21,22,23,24]. A smart way to use more leads, marginally compromising patient comfort, could be by embedding the electrodes into clothes. Dry capacitive electrodes are highly suitable for this kind of application. In order to use capacitive electrodes, new electronic interfaces should be developed to match the application requirements. The two main requirements are electronic interface high impedance and EMI rejection.

In this study, the possibility of recording multiple-lead ECG using dry capacitive electrodes with a custom-developed portable device was investigated to explore the feasibility of a wearable ECG device with electrodes embedded into clothes. The performances of an electronic interface proposed by Chi et al. [25] and others [26,27] were evaluated by characterizing it with simulations and electrical tests in order to deeper investigate its performances. Moreover, the studied electronic interface was implemented into a custom realized portable device, and in order to verify the effectiveness of the system, a comparison between signals obtained using the developed device, with dry capacitive electrodes, and a commercial heart rate front-end IC, with standard wet electrodes, was performed.

The novelty of this study is the deeper understanding of the studied electronic interface behavior, with respect to published results. This goal was achieved by characterizing the electronic interface in detail using simulations and electrical tests. An opportune experimental setup was designed to perform the measurements with experimental conditions as close as possible to real applications; that is, an “ECG T-shirt” with an embedded capacitive electrode.

According to the obtained results, a specific circuit optimization method was proposed. Moreover, these results could be exploited by numerous real applications, such as drivers’ safety [21,28,29,30,31,32,33], remote monitoring of the elderly [34,35,36,37], sleep monitoring [20,38,39,40,41,42], human identification [43,44,45,46] and emotion recognition [47,48,49,50].

## 2. Materials and Methods

Biopotential signals, such as those generated by the electrical activity of the heart, are usually measured with a differential approach using voltage amplifiers with at least two basic requirements: input impedance, which should be as high as possible in order to match the electrode impedance, and the current flowing between the body and the amplifiers, which should be as low as possible for safety reasons.

Biopotential measurements, such as ECG, are very sensitive to electromagnetic interference (EMI). One of the most experienced EMI is caused by the coupling between the patient’s body and the power lines, resulting in an applied common-mode voltage (V_cm_) on the electrodes.

By performing differential biopotential measurement and improving the common-mode rejection (CMR) of the system, it is possible to contain this phenomenon. Differential measurement can be obtained by applying the biopotentials to the input stage of a classic instrumentation amplifier (INA) topology.

EMI generates an I_cm_ flowing through the patient’s body from the sinusoidal power-line source (120 V/60 Hz or 230/50 Hz) through coupling capacitances to the ground. In order to perform an effective ECG measurement, the patient’s body has to be grounded with the ECG device. This grounding connection contributes to the rise in the V_cm_ primarily because of its resistance. As a consequence, it is important to reduce the impedance of the grounding electrode or to compensate for the EMI-generated current. This result can be achieved using a driven right leg (DRL) electrode [51]. It is commonly applied to the right leg, but it can be applied elsewhere on the patient’s body.

The DRL electrode senses the common-mode voltage on the body, giving feedback to the patient’s body. This negative feedback reduces V_cm_ to µV values [52].

This study investigates the potentialities of a suitable circuit topology, originally proposed by Chi et al. [25], to enhance its applicability via a deeper characterization, which should allow exploiting this strategy for innovative devices.

**Figure 1:**
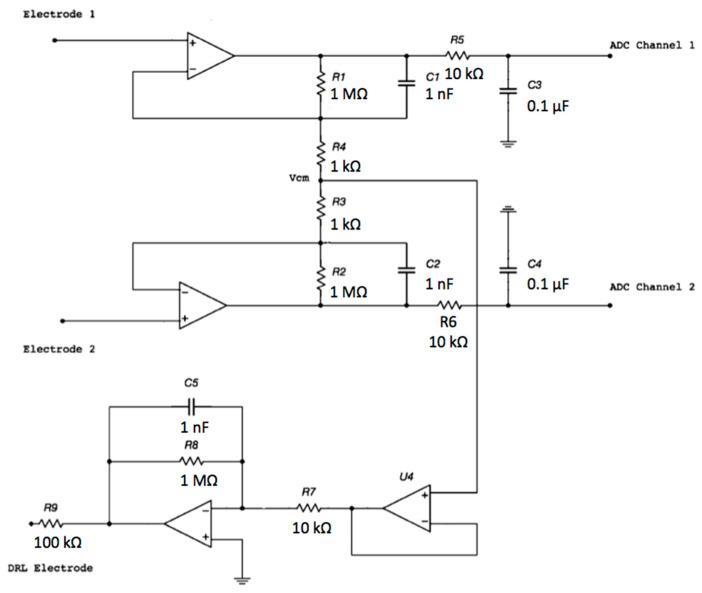
Schematic of the electronic interface. Each electrode is connected to the buffering stage of an instrumentation amplifier (INA) configuration. The buffered and filtered output voltages will be acquired by two ADC (Analog To Digital Converter) channels with respect to the ground. The common-mode voltage is connected to the driven right leg (DRL) electrode and is rejected.

The proposed electronic system uses two sensing capacitive electrodes and a DRL capacitive electrode, as shown in Figure 1.

The capacitive electrodes can be placed on the patient’s clothes (e.g., a cotton shirt) and not be in contact with the skin. Therefore, from now on, we will refer to the system as a contactless ECG device.

Each electrode is an input of the buffering stage of a differential amplifier based on the topology of a 3-input amplifier. The DRL is made of an inverting opamp and a buffering stage.

**Figure 2:**
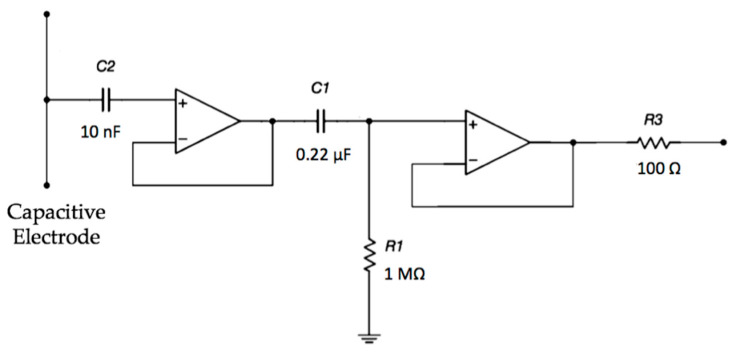
Schematic of the electrode interface. A double buffering stage is implemented in order to achieve a very high input impedance.

The electrodes are active and use amplifiers in order to achieve a very high input impedance, as shown in Figure 2. A high-pass filtering stage, formed by R_1_ and C_1_, is used to reduce the signal artifacts.

The CMR and the frequency response of the electronic interface of the contactless ECG were characterized by simulating the circuit in MULTISIM (National Instruments). The circuit was implemented on a PCB and tested. According to the obtained results, optimization of the circuit is proposed.

Moreover, we developed a portable device based on the presented electronic layout. The output signals of the electronic interface are acquired using the 12-bit SAR ADCs of the ESP32 microcontroller (ESPRESSIF SYSTEMS, Shanghai) with a sampling frequency of 250 Hz. The ESP32 microcontroller is equipped with an internal Bluetooth module; thus, it is capable of transmitting digitized signals through a wireless connection to a PC or smartphone. The device is battery operated.

**Figure 3:**
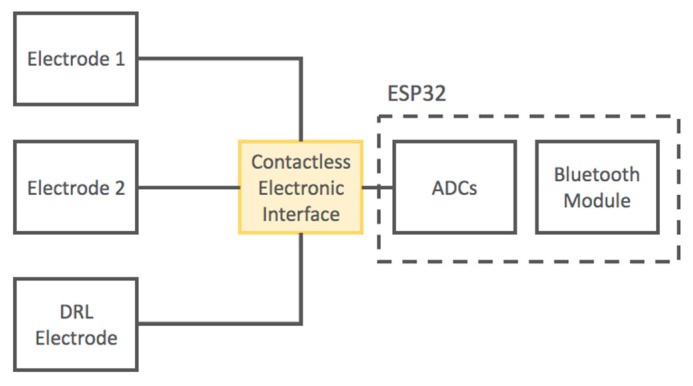
Block diagram of the developed contactless electrocardiogram (ECG) device.

A schematic of the developed device is reported in Figure 3.

**Figure 4:**
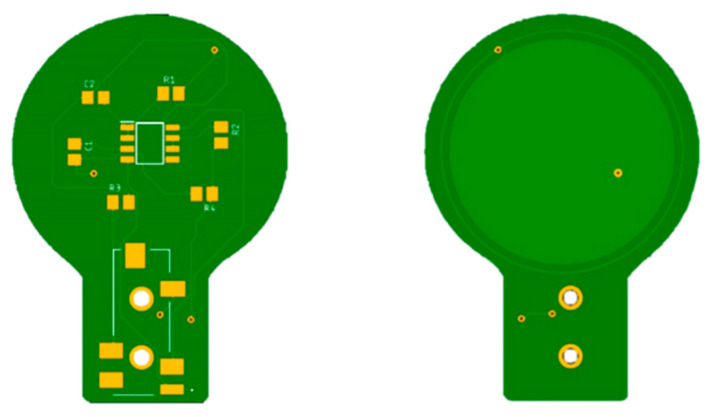
PCBs of the capacitive electrodes. The armature plate is placed in the bottom layer of the board under an insulating layer. It is made of copper and has a radius of 3 cm.

Each electrode is connected to the electronic interface using a 4-pole audio cable. The armature plate of the electrodes was implemented using directly the bottom layer of the PCBs, as shown in Figure 4.

To evaluate the effectiveness of the contactless ECG device, we performed a comparison between signals obtained using this device and those obtained using an AD8232, a fully integrated single-lead ECG front end by Analog Devices, using standard wet electrodes. The AD8232 is an integrated signal conditioning block for portable ECG devices and other biopotential measurement applications. It implements a filter and a differential amplification stage. To improve the common-mode rejection of the IC, it includes an additional amplifier for the right leg drive [53].

**Figure 5:**
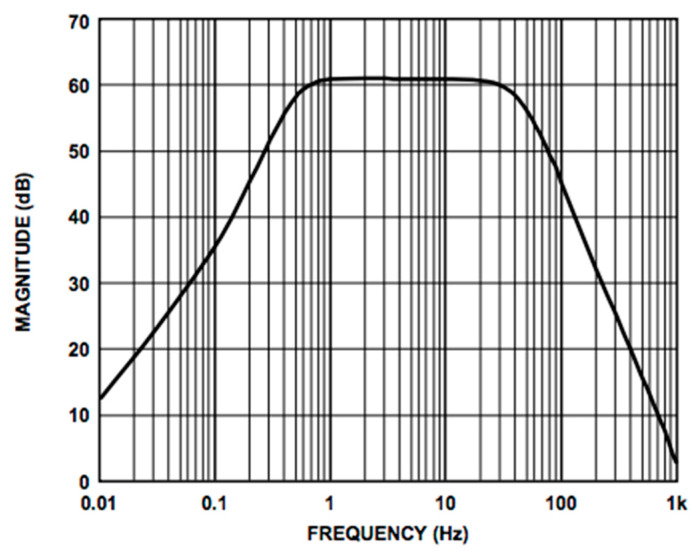
Frequency response of the AD8232 implemented in the “Cardiac Monitor Configuration.” The bode plot shows a band-pass behavior in the range of frequencies between 0.5 and 40 Hz and a gain of about 60 dB (1000 *V*/*V*) (the bode plot is extracted from the AD8232 datasheet [53]).

We used the AD8232 in the “Cardiac Monitor Configuration,” as suggested by the IC’s application note [53]. This configuration is designed to perform ECG measurements with minimal distortion. The AD8232 is configured with a 0.5 Hz two-pole high-pass filter followed by a 40 Hz two-pole low-pass filter, and the total system gain is about 60 dB. A DRL is used to improve common-mode rejection; thus, the electronic approach is the same as the developed contactless ECG device. The frequency response of the system is reported in Figure 5.

The output signals from the AD8232 are acquired using the ADCs of the ESP32 microcontroller with a sampling frequency of 250 Hz. The ESP32 microcontroller is equipped with an internal Bluetooth module; thus, it is capable of transmitting digitized signals through a wireless connection to a PC or smartphone. The device is battery operated.

**Figure 6:**
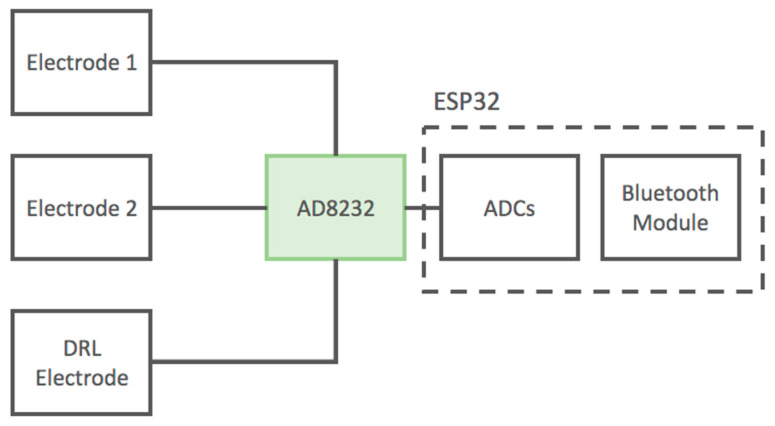
Block diagram of the developed ECG device based on the AD8232 IC. The device uses standard wet adhesive electrodes.

The scheme of the developed device is reported in Figure 6.

In order to avoid interference between cables and electrodes of the two devices, ECG measurements were performed sequentially, placing the electrodes in the same position with the same experimental condition. The position of the electrodes implements Lead I of Einthoven’s triangle.

Several measurements were performed on a single healthy subject at rest. This is because our primary goal in this study was to evaluate the feasibility and effectiveness of the studied electronic interface; even one subject was sufficient. Clearly, further studies on a larger population will be necessary in order to clinically test the developed device.

**Figure 7:**
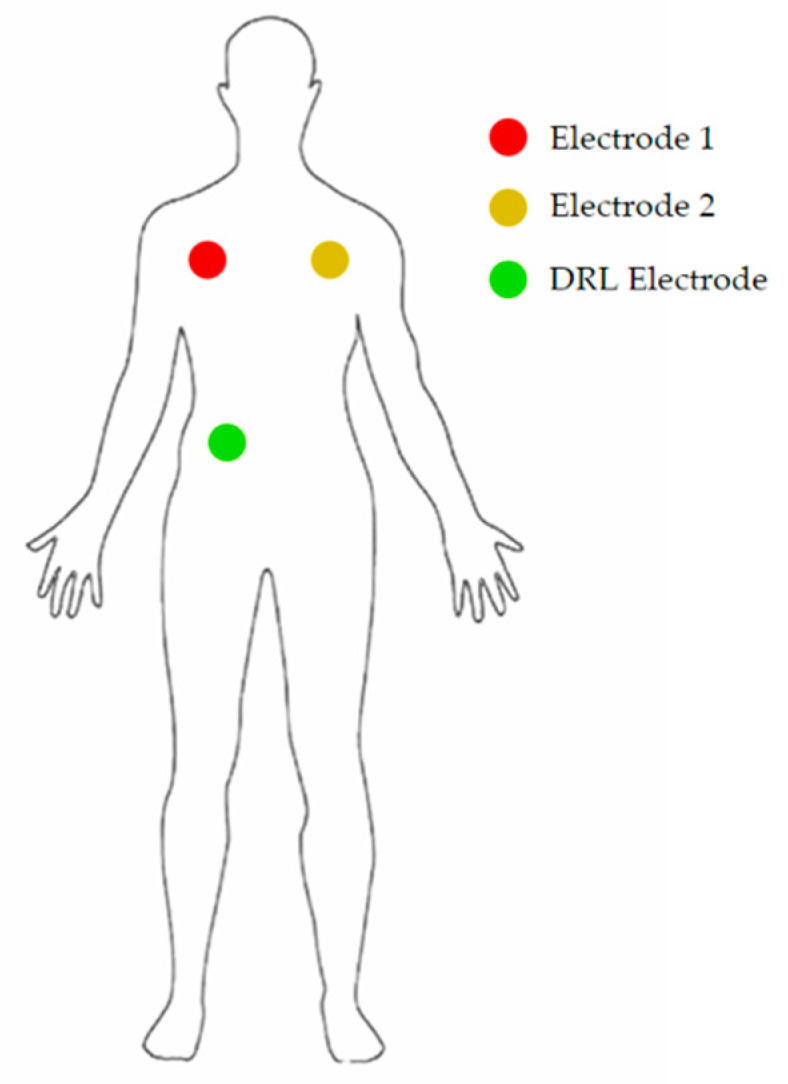
Position of the 3 electrodes on the body of the patient.

The electrodes were placed as shown in Figure 7.

## 3. Results

The electronic interface of the contactless ECG device was characterized. The frequency response of the electrodes was investigated.

**Figure 8:**
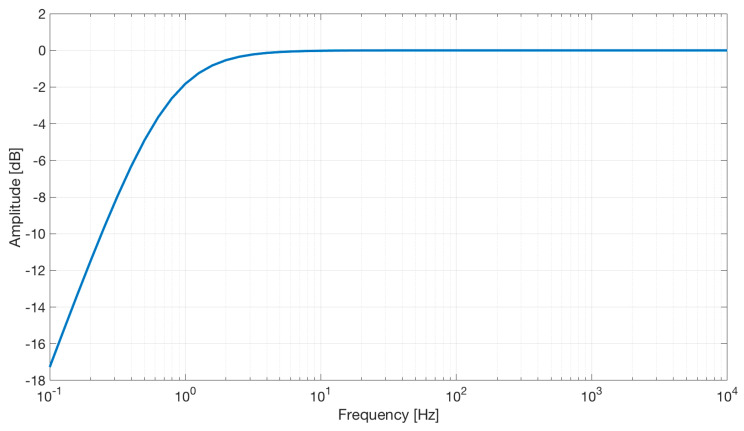
Frequency response of the active capacitive electrodes.

The electrical scheme of the electrode is reported in Figure 2. The simulation, reported in Figure 8, shows a first-order high-pass characteristic with a cut-off frequency of about 0.7 Hz and no gain.

It is useful to introduce a high-pass filtering stage to reduce the signal artifacts caused by electrodes or body movements, such as thoracic expansion during breathing.

**Figure 9:**
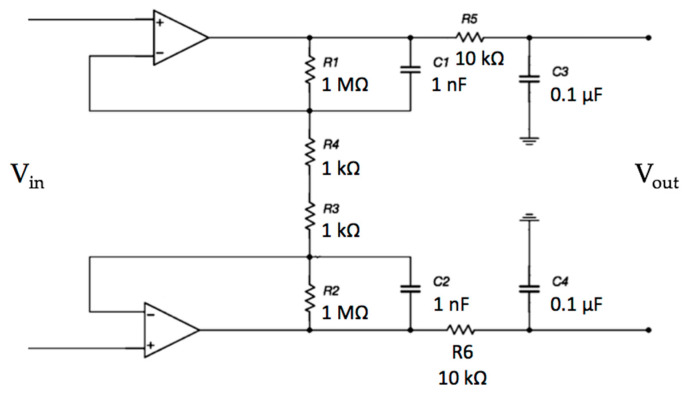
Schematic of the differential stage. In this stage, the input signal is amplified and filtered.

The frequency response of the differential opamp stage was investigated. The electrical scheme is reported in Figure 9.

**Figure 10:**
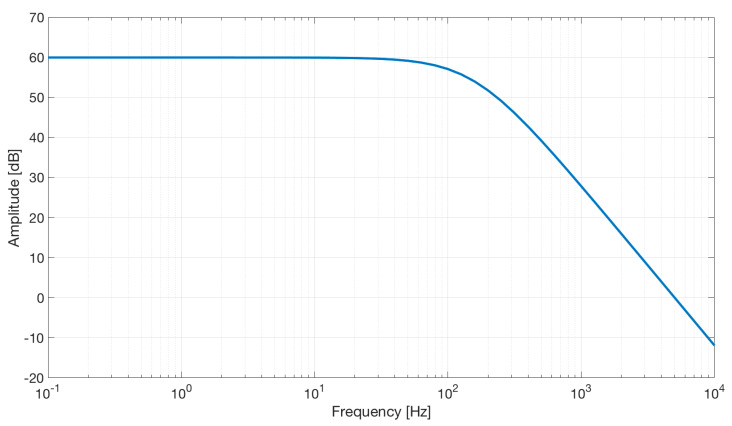
Frequency response of the differential stage.

The simulation shows a second-order low-pass characteristic of the differential amplifier with a gain of 60 dB and a bandwidth of about 100 Hz (see Figure 10).

**Figure 11:**
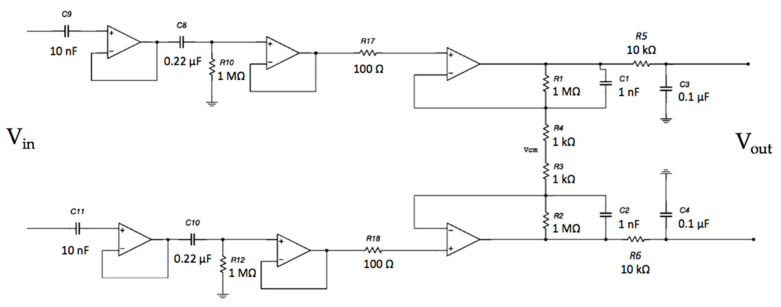
Schematic of the capacitive active electrodes coupled with the differential stage.

The system frequency response of the electrodes coupled with the differential stage was investigated. The complete circuit is shown in Figure 11.

**Figure 12:**
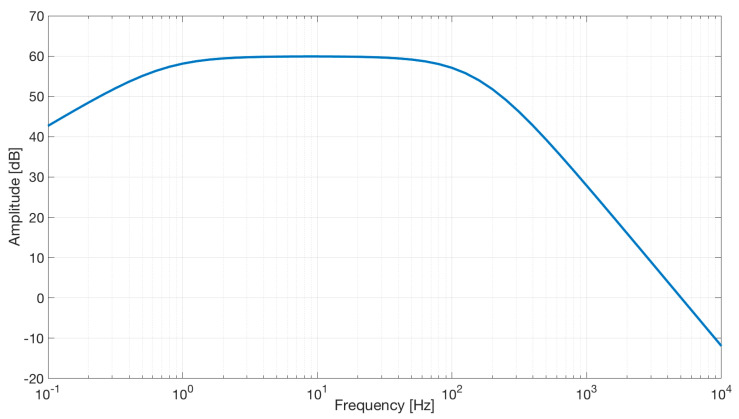
Frequency response of the capacitive active electrodes coupled with the differential stage. The bode plot shows a band-pass behavior in the range of frequencies between 0.7 and 100 Hz and a gain of about 60 dB.

As expected, simulation results (see Figure 12) show a band-pass characteristic with a gain of 40 dB.

**Figure 13:**
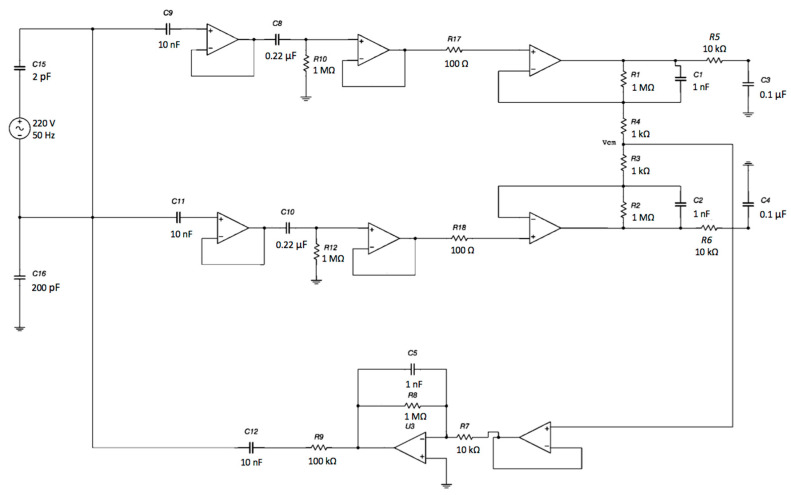
Schematic of the complete system: capacitive electrodes, differential stage and DRL electrode. The electromagnetic interference from the power line was modeled as a sine voltage source coupled to the electrodes through a 2 pF capacitor. Moreover, the patient’s body was also coupled to the ground through a 200 pF capacitance [51].

The voltage output of each channel with respect to the ground will exhibit a common-mode voltage gain equal to 1.0. Obviously, by performing a differential measurement between the output voltage of Channel 1 and the output voltage of Channel 2, the V_cm_ will theoretically disappear (in a real scenario, it is a small voltage). Using a DRL circuit, it is possible to further increase the CMR of the system. Different gains of the DRL stage were simulated to study its feedback effect on V_cm_. The schematic is shown in Figure 13.

**Figure 14:**
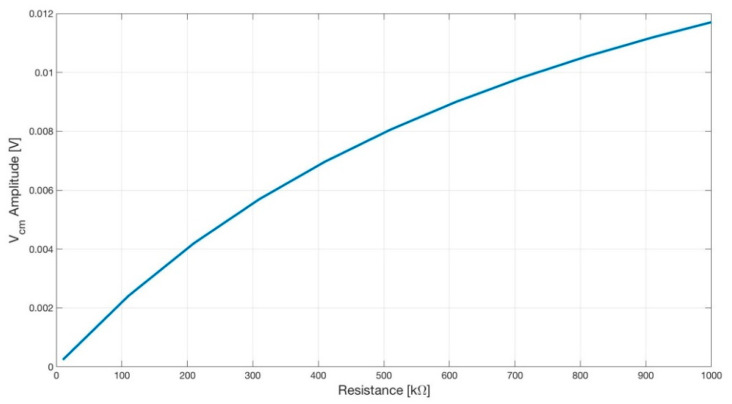
Plot showing the amplitude of the common-mode voltage V_cm_ versus the input resistance R7 (see Figure 15) of the DRL opamp. As expected, by lowering the gain of the DRL, the amplitude of the V_cm_ increased.

The simulation results are shown in Figure 14.

**Figure 15:**
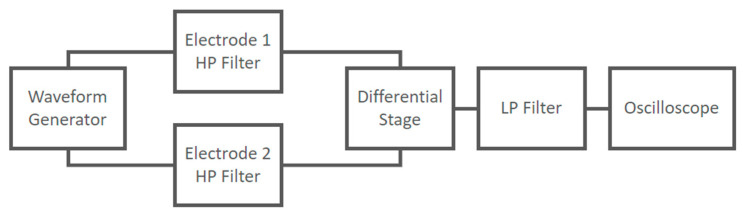
Experimental setup at block scheme level used to characterize the frequency response of the capacitive electrodes coupled with the differential stage.

The implemented circuit shown in Figure 11 was tested to verify its frequency response using a Lecroy Wave Station 3082 Waveform Generator as input and recording the output signals using a Lecroy HDO6054-MS Oscilloscope. The experimental setup at the block scheme level is shown in Figure 15.

**Figure 16:**
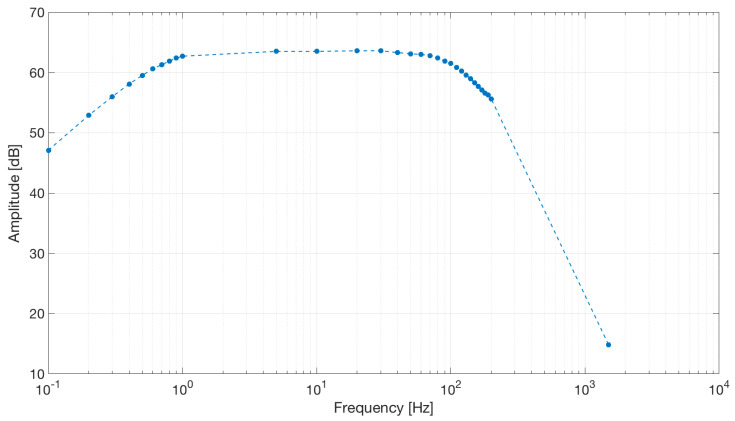
Frequency response of the capacitive active electrodes coupled with the differential stage. The band-pass behavior shown by the simulation was confirmed by the electrical tests. Dots represent experimental data.

The frequency response of the circuit is shown in Figure 16.

The output signals were acquired using the internal 12-bit SAR ADCs of the ESP32 microcontroller and transmitted using Bluetooth to a PC. The sampling frequency was 250 Hz.

**Figure 17:**
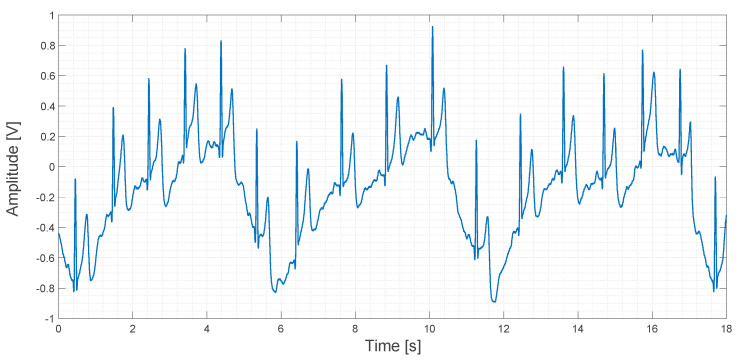
Output signal obtained using the contactless ECG device.

About 20 s of recorded data obtained using the contactless ECG device are shown in Figure 17.

In Figure 17, a sinusoidal pattern is clearly visible. It is caused by the movement of the rib cage during breathing. Even if this pattern is a sort of signal artifact, it could be used to determine the respiratory rate during an ECG measurement. In any case, this kind of low-frequency artifact can be filtered both in the analog and in the digital domain by applying a high-pass filter. In the original study, a compression vest was used to keep the electrodes in fixed positions and to mitigate this kind of artifact, but we think that this approach is not very comfortable for patients. In contrast, we used an elastic band in order to apply the same compression that a cotton T-shirt would apply. One of our aims in this study is to explore the feasibility of the embedding of the electrodes into clothes. In fact, a smart way to use several electrodes for a long period of time, marginally compromising patient comfort, could be, for example, by embedding the electrodes directly into a T-shirt.

**Figure 18:**
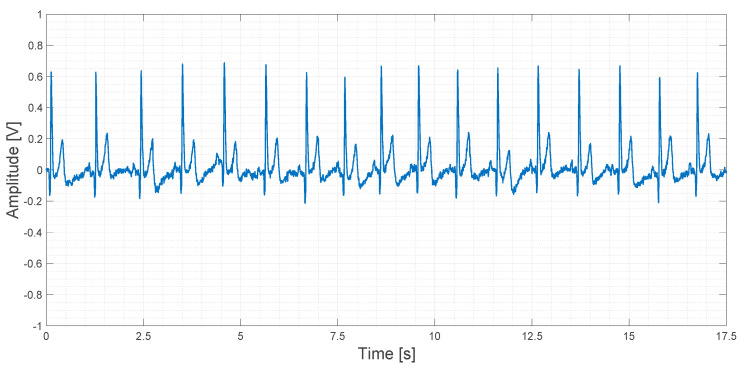
Output signal obtained using the AD8232 device.

About 20 s of recorded data obtained using the AD8232 device are shown in Figure 18.

In Figure 18, a slightly higher noise floor can be observed. No artifacts are shown.

**Figure 19:**
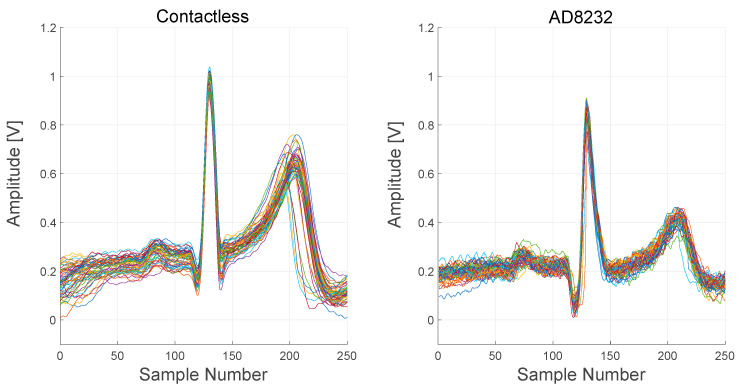
Comparison between the contactless device and AD8232 device signals. About 60 PQRST complexes were extracted from the raw output signals and overlapped in a single plot for each device.

In Figure 19, signals recorded from both the contactless device and the AD8232-based device are reported. In order to better visualize the difference between the signals, several PQRST complexes, extracted from the raw output signals, are plotted.

ECG complexes, extracted from the two devices, are quite comparable but not identical. The timings of intervals and segments of the complexes are the same, but the relative amplitudes of the R peak and the T wave are different. Moreover, S peaks are not visible in the AD8232 complexes.

## 4. Discussion and Conclusions

We simulated, implemented and tested an electronic interface for ECG devices. The results obtained from simulations show the ability of the electronic interface to amplify differential biopotentials and to reject common-mode signals produced by EMI in an effective way. The results obtained from the electrical testing on the implemented PCB confirm these capabilities. Further adjustments can be applied in order to mostly optimize the response of the system at low frequencies.

The studied interface can be used with a capacitive electrode that can be placed on the clothes of the patient. This contactless ECG device is more comfortable for long-term monitoring instead of standard ECG devices using adhesive wet electrodes. The circuit topology allows the implementation of the electronic interface with very low costs; in fact, unlike many other electronic interfaces for ECG devices, it does not use expensive Instrumentation amplifiers. Moreover, the topology is highly modular, allowing the use of a flexible number of electrodes.

To evaluate the effectiveness of the contactless ECG device, we performed ECG measurements on a healthy subject. The biopotentials were acquired using both the studied electronic interface and a commercial fully integrated single-lead ECG front end by an analog device. Both electronic interfaces use an approach based on differential amplification, filtering and DRL electrodes in order to obtain a high signal-to-noise ratio. The first electronic interface makes use of capacitive electrodes, and the second interface uses standard wet Ag/AgCl electrodes.

The obtained measurements are quite comparable in terms of the amplitude and shape of the recorded signal. The ECG traces, recorded using a contactless device, present a satisfying signal-to-noise ratio. Moreover, the acquired signals present all the features needed for a clinical evaluation: P waves, QRS complex and T wave. Contactless ECG traces showed a sinusoidal pattern caused by the movement of the rib cage during breathing. This artifact could be used to determine the respiratory rate of the patient, obtaining one more useful piece of information during the ECG measurement. The characterization of the electronic interface behavior in the frequency domain highlights how optimization of the filtering stages could decrease this kind of artifact.

Moreover, capacitive electrodes are sensitive to nearby charge displacements. As was expected, during the tests, we experienced signal artifacts of the output signal caused by body movements, in particular arm movements and, in a minor way, movements of the head and shoulders. We think that before testing the system on a person in motion (e.g., walking, running), further investigations are needed to understand how to reduce signal interferences generated by movements of the upper body.

It is important to highlight that although we tested the contactless ECG device implementing only two electrodes and a DRL grounding electrode in a single-lead configuration, it can also be used in a multiple-lead configuration with more than two electrodes.

In further studies, we would like to implement and test the contactless ECG device with at least three leads in order to record standard and augmented limb leads. With more electrodes, it would be possible to record a complete 12-lead ECG.

Thus, the use of a greater number of electrodes allows the recording of information content greater than those obtainable with wearable devices (e.g., smartwatches) currently available on the market. The implementation of more leads and the recording of more informative signals will allow going beyond mere arrhythmia diagnosis. In any case, further studies will be needed to evaluate the possibility of using ECG recorded using a multilead contactless ECG device to provide a diagnosis of other cardiac disorders.

Obviously, it will be less comfortable to measure an ECG using several electrodes placed on the body instead of a smartwatch, but huge improvements can be performed with regard to the implementation of the electrodes. As the capacitive electrodes do not require direct contact with the patient’s skin, they could be embedded inside the clothes; for example, in the fabric of a T-shirt using flexible electronics. Therefore, in further studies, we would also like to optimize the implementation of the electrodes in order to make the contactless ECG device more comfortable and wearable.

## Data Availability

Data are available upon reasonable request.
